# Discordance between humoral and cellular immune responses to cytomegalovirus infection in CMV seropositive patients awaiting lung transplantation

**DOI:** 10.3389/fimmu.2024.1445553

**Published:** 2025-01-22

**Authors:** Meritxell Boada-Pérez, Cristina Berastegui, Marta Erro, Piedad Ussetti, Elena Crespo, Laura Donadeu, Oriol Bestard, Gabriel Anguera, Amparo Solé, Ricardo Ponz Mir, Brian Molloy, Eva Revilla-López, Víctor Monforte, Susana Gómez-Ollés

**Affiliations:** ^1^ Department of Medicine, Universitat Autònoma de Barcelona, Barcelona, Spain; ^2^ Department of Pulmonology, Vall d’Hebron Research Institute, Barcelona, Spain; ^3^ Vall d’Hebron for Solid Organ Transplantation Research Group, Vall d’Hebron Research Institute, Vall d’Hebron Barcelona Hospital, Universitat Autònoma de Barcelona, Barcelona, Spain; ^4^ Lung Transplant Unit, Pulmonology Department, Vall d’Hebron University Hospital, Barcelona, Spain; ^5^ Centro de Investigación Biomédica en Red de Enfermedades Respiratorias (CIBERES), Instituto de Salud Carlos III, Madrid, Spain; ^6^ Department of Pulmonary Medicine, Puerta de Hierro Majadahonda University Hospital, Majadahonda, Madrid, Spain; ^7^ Laboratory of Nephrology and Transplantation, Vall d’Hebron Research Institute, Vall d’Hebron Barcelona Hospital, Universitat Autònoma de Barcelona, Barcelona, Spain; ^8^ Kidney Transplant Unit, Nephrology Department, Vall d’Hebron University Hospital, Vall d’Hebron Barcelona Hospital, Universitat Autònoma de Barcelona, Barcelona, Spain; ^9^ Lung Transplant Unit, Hospital Universitari i Politècnic La Fe, Valencia, Spain; ^10^ Medical Affairs Department, Merck Sharp & Dohme (MSD) Spain, Madrid, Spain

**Keywords:** ELISPOT, infection risk, immunocompromised, pre-transplant, serology, CMV cell-mediated immunity, humoral immune response

## Abstract

**Introduction:**

Risk stratification for CMV infection in lung transplantation (LT) currently relies on determining donor and recipient CMV IgG before transplantation. However, it has been observed that some patients who test positive for CMV-specific humoral response before kidney transplantation (KT) exhibit a weak or absent CMV-specific cellular response. The significance of this observation in LT is still unknown.

**Methods:**

This prospective, multicenter, observational study evaluated the agreement between CMV IgG serology and specific cell-mediated response (specific T cell Enzyme-Linked ImmunoSpot Assay, ELISPOT, against CMV pp65 and IE-1 antigens) in 121 patients on the waiting list for LT.

**Results:**

One hundred and four (86%) patients were seropositive for CMV. Discordant humoral and cellular immunologic responses were observed, 29% of seropositive patients had a weak ELISPOT response to IE-1 and 39% to pp65. In 22% of seropositive patients, there was a weak or no response to both antigens. All seronegative patients did not respond to either antigen.

**Conclusions:**

Therefore, over 20% of CMV seropositive LT candidates showed weak CMV-specific cellular immune responses despite detectable serological memory against CMV. This may be important in assessing the risk of developing a CMV infection after transplantation.

## 1 Introduction

Cytomegalovirus (CMV) is one of the most common and clinically relevant opportunistic infections following a solid organ transplant (SOT) and increases morbidity and mortality (1–6). Moreover, CMV exerts an immunomodulatory activity in the host, increasing the risk of other infections, acute rejection (7–9) and/or other forms of chronic graft dysfunction (10–12).

The incidence of CMV infection after transplantation differs greatly depending on the type of transplanted organ, the serostatus of both the donor (D) and recipient (R) (13), and the use of prevention strategies (14). In the case of lung transplantation (LT), the incidence of CMV disease is higher than in other SOT, varying from 8% to 55% depending on the type and duration of prophylaxis (15–18). Due to its negative impact, universal antiviral prophylaxis with intravenous ganciclovir and oral valganciclovir is an established standard of care for patients undergoing LT. Anti-CMV prophylaxis is usually administered for 6 or 12 months after LT, depending on D and R serology status, although the optimal length remains unclear (19). CMV prophylaxis effectively reduces the risk of CMV disease, but a long-lasting prophylaxis therapy is associated with side effects that often lead to treatment withdrawal, mainly due to leukopenia.

The CMV serologic IgG status of the D and R prior to transplantation is usually assessed to stratify the risk of post-transplant infection and to individualize antiviral prophylaxis (20). In general, D+/R- solid organ transplant recipients are considered at the highest risk of CMV infection or disease, followed by D+/R+ and D-/R+ recipients, who are at intermediate risk, while D-/R- recipients have the lowest risk of CMV infection or disease (6, 13, 15). However, differences in CMV disease risk between D+/R+ and D-/R+ have also been observed, with evidence indicating that D+/R+ recipients develop CMV infection more frequently than D-/R+ recipients over 24 months after transplantation (21). It is generally assumed that CMV-seropositive patients have pre-existing immunity that helps control subsequent replication episodes. Nevertheless, a non-negligible percentage of R+ developed CMV infection (21). For instance, a study with LT recipients showed that 18 months after transplantation, 25% of R+ recipients developed CMV infection and 15% developed CMV disease (15). Currently, although the importance of CMV-specific cell-mediated immunity (CMV-CMI) is known, in clinical practice, risk stratification is solely based on the humoral response. However, in kidney transplantation (KT), few studies have explored the utility of CMV-specific cell-mediated immunity (CMI) for stratifying the risk of CMV infection after transplantation. These studies have reported that patients with discordant results between humoral and cellular CMV-specific responses and with a negative CMI before KT were at higher risk of CMV infection after transplantation (22–24). These studies demonstrated the usefulness of assessing CMV-specific cellular response in combination with serostatus for guiding prophylaxis. While it is true that this has been demonstrated in kidney transplant candidates, this does not necessarily imply that the same discordance will occur in lung transplant candidates due to the different underlying diseases and treatments these patients receive before transplantation. Therefore, in this study we aimed to assess the agreement between CMV-specific humoral and cellular responses, analyzed by IgG serology and T cell Enzyme-Linked ImmunoSpot Assay (ELISPOT), respectively, in a LT cohort.

## 2 Materials and methods

### 2.1 Study design and population

It was a prospective, observational, multicenter study where 165 patients from 3 LT waiting lists were included (**Table 1**). Patients were recruited from October 2019 to July 2022. Samples were obtained from 121 patients and both, CMV IgG serology and CMV T cell ELISPOT, assays were performed.

**Table 1:** Clinical and demographic characteristics from those 121 patients with specific CMV humoral and cellular responses assessment.

	Overall (n=121)	Positive CMV IgG (n=104)	Negative CMV IgG (n=17)	p^1^
Age, mean years (SD)	56.4 (9.3)	57.0 (9.4)	52.8 (8.5)	0.0303
Gender, n (%)				
Male	72 (59.5)	64 (61.5)	8 (47.1)	0.2595
Female	49 (40.5)	40 (38.5)	9 (52.9)	
Blood group, n (%)				
A	29 (24)	27 (26)	2 (11.8)	0.0343^(c)^
B	14 (11.5)	13 (12.5)	1 (5.9)	
A/B	3 (2.5)	3 (2.9)	0 (0.0)	
O	75 (62)	61 (58.7)	14 (82.4)	
Underlying lung disease, n (%)				
Interstitial lung disease (ILD)	47 (38.8)	41 (39.4)	6 (35.3)	0.5910^(c)^
Chronic obstructive pulmonary disease (COPD)-emphysema	47 (38.8)	39 (37.5)	8 (47.1)	
Cystic fibrosis	7 (5.8)	6 (5.8)	1 (5.9)	
Bronchiectasis	3 (2.5)	3 (2.9)	0 (0.0)	
Pulmonary hypertension	2 (1.7)	1 (1.0)	1 (5.9)	
Underlying conditions				
Hypertension	28 (23.1)	25 (24.0)	3 (17.6)	0.7595^(f)^
Dyslipidemia	25 (20.7)	23 (22.1)	2 (11.8)	0.5198^(f)^
Diabetes	14 (11.6)	14 (13.5)	0 (0.0)	0.2144^(f)^
Hepatitis B	3 (2.5)	3 (2.9)	0 (0.0)	1.0000^(f)^
Autoimmune disease	2 (1.7)	2 (1.9)	0 (0.0)	1.0000^(f)^
Hepatitis C	1 (0.8)	1 (1.0)	0 (0.0)	1.0000^(f)^
None	26 (21.5)	22 (21.2)	4 (23.5)	0.7595^(f)^
Unknown	4 (3.0)	3 (2.6)	1 (5.9)	0.4588^(f)^
Risk factors				
Current smoker, n (%)				
Yes	0 (0.0)	0 (0.0)	0 (0.0)	0.7138
No	45 (37.2)	38 (36.5)	7 (41.2)	
Former smoker	76 (62.8)	66 (63.5)	10 (58.8)	
Total years smoking (only former smokers), mean (SD)	12.6 (11.2)	12.8 (11.1)	11.2 (12.8)	0.3573

^1^Comparisons between groups: Mann–Whitney U-test for continuous variables; Chi-squared test for categorical variables. ^1^Comparisons between groups: Chi-square test (c) or Fisher’s exact test (f). SD, standard deviation.

Patients were over 18 years old and written informed consent was obtained from all included patients.

Data was rigorously collected and included in a centralized electronic Case Report Form (eCRF) and audited by an external Contract Research Organization (CRO) to guarantee data fidelity.

All procedures followed in this study were in accordance with the ethical standards of the three participating hospitals’ ethics committees and with the Declaration of Helsinki (2013). Before the initiation of the study, it was approved by the Ethics Committee of Hospital Universitario Puerta del Hierro Majadahonda (8/2019), and then local ethics committee approval was obtained from the other two participating hospitals.

### 2.2 ELISPOT assay determination and categorization

Blood samples were collected in three VACUETTE lithium heparin tubes^®^ before LT. Peripheral blood mononuclear cells (PBMCs) were isolated from patients’ peripheral blood by Ficoll-Paque ^®^ density gradient centrifugation, stored at -80°C for 24h and then frozen in liquid nitrogen until they were used in functional analyses. The assessment of CMV specific T cell activity against two major immunogenic CMV antigens, immediately-early 1 (IE-1) protein and phosphoprotein 65 (pp65), was performed with T-SPOT^®^.CMV (Oxford Immunotec Ltd, Abingdon, UK) according to previous standard operating procedures (25–27), using previously isolated PBMCs. Briefly, 3 x 10^5^ PBMCs (100µL) were stimulated in duplicate with a CMV antigen peptide pool (1µg/mL) for 18 hours. Then, IFN-γ producing cells were detected using an anti-human IFN-γ antibody conjugated to alkaline phosphatase. Followed by the addition of a soluble substrate, the product precipitate and it is quantified by counting spots semiautomatically with an ELISPOT reader (AID ELISPOT Reader HR, 4^th^ generation).

CMV specific cellular activity was categorized based on previous publications in KT. Cutoff points for IE1 and pp65 were established at 25 spots/3x10^5^ and 130 spots/3x10^5^ cells, respectively, with lower or equal values being categorized as negative results and higher values as positive (25, 26).

The classification of CMV-specific cellular response was based on the one used in previous KT studies (25–28). Strong CMV cellular response was considered when there is a positive ELISPOT result to both peptides (IE-1 ELISPOT >25 spots/3x10^5^ cells and pp65 ELISPOT >130 spots/3x10^5^ cells); intermediate response when there is a negative result to one of the two peptides (IE-1 ≤25 or pp65 ≤130 spots/3x10^5^ cells), and weak response when ELISPOT results for both peptides is negative (IE-1 ELISPOT ≤25 spots/3x10^5^ cells and pp65 ELISPOT ≤130 spots/3x10^5^ cells).

### 2.3 CMV serologic IgG status

Blood was drawn in a BD Vacutainer^®^ serum tube. Pretransplant CMV serological status in patients awaiting LT was assessed through Electrochemiluminescence immunoassay (ECLIA).

### 2.4 Statistical analysis

Continuous variables were represented as mean ± standard deviation (SD) or median ± interquartile range (IQR). Categorical variables were described as frequencies and percentages. The normality of distributions was evaluated before performing statistical analysis in order to determine the most suitable test for each case. Chi-Square or Fisher’s exact tests were used for comparisons between qualitative variables, while for continuous ones t-test for two independent variables/ANOVA test (>2 categories) or Mann Whitney/Kruskal Wallis U test were performed. To evaluate concordance between CMV serology and ELISPOT, Cohen’s Kappa test was performed with the confidence interval (CI) of 95%. Cohen’s Kappa results are categorized as: <0.01 poor, 0.01-0.20 slight, 0.21-0.40 fair, 0.41-0.60 moderate, 0.61-0.8 substantial and 0.81-1.00 excellent concordance (29). Statistical significance was established at p ≤ 0.05. All analyses were performed through the SAS version 9.4 statistical software.

## 3 Results

### 3.1 Patient characteristics

From those 121 patients with specific CMV humoral and cellular responses assessment, 104 (86%) patients were CMV seropositive patients and 17 (14%) were seronegative. Seronegative patients were younger (*p*=0.030) and the most frequent blood group was O (*p*=0.034). No statistically significant differences were observed between both groups in other demographic and clinical variables (**Table 1**).

### 3.2 CMV-specific cellular response

**Figure 1:**
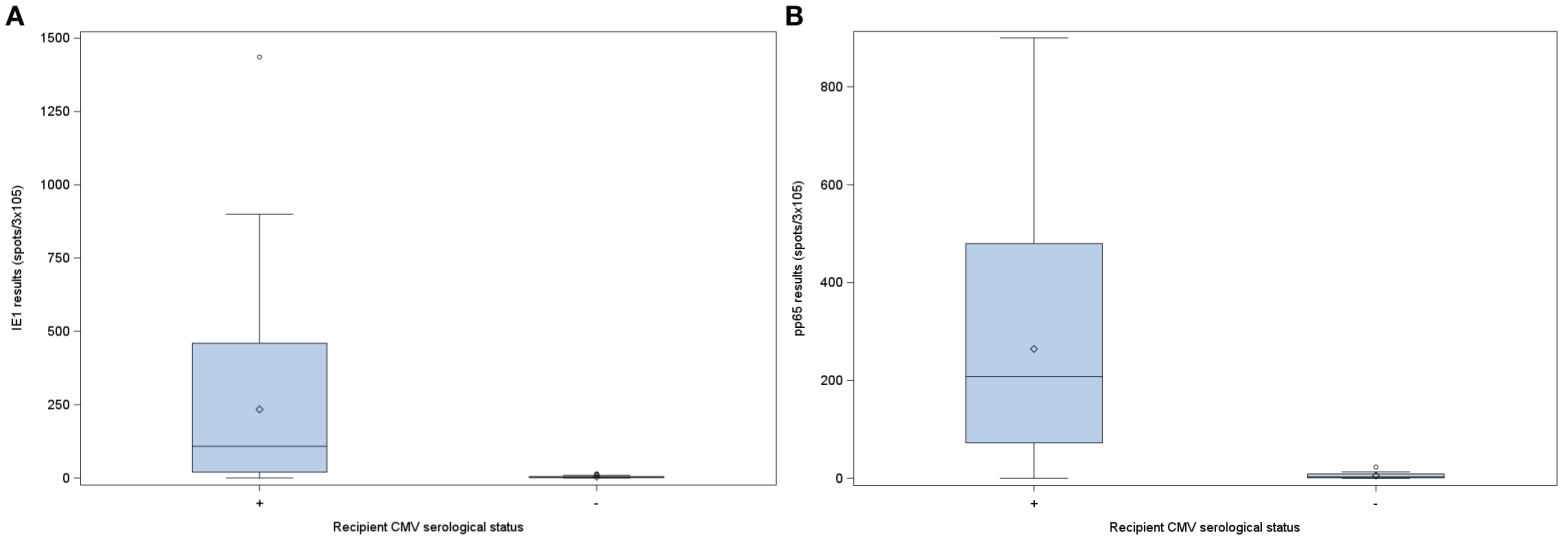
Pre-transplant CMV ELISPOT results categorized by recipient CMV serological status prior to transplantation. **(A)** ELISPOT responses upon stimulation with IE-1. **(B)** ELISPOT responses upon stimulation with pp65. ELISPOT, Enzyme-Linked Immunosorbent Spot assay; IE-1, immediately-early 1 protein; pp65, phosphoprotein 65; CMV, cytomegalovirus.

**Figure 2:**
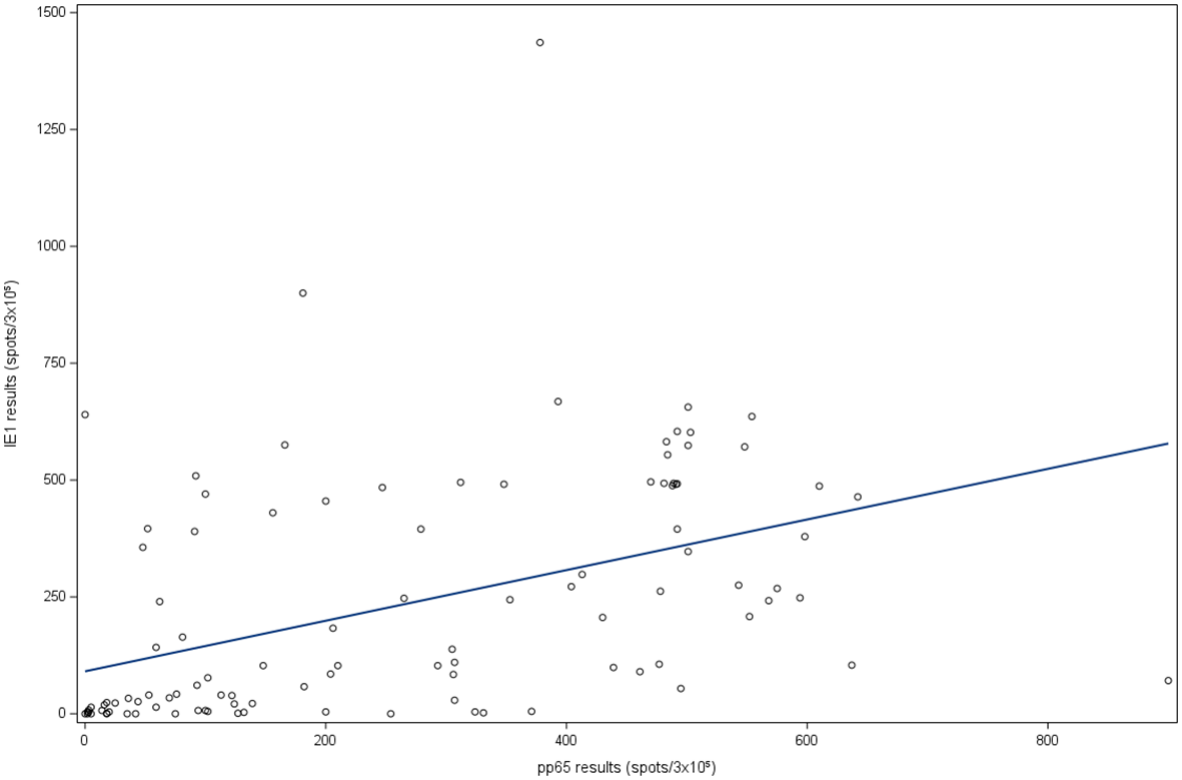
Linear correlation between IE-1 and pp65. IE-1: immediately-early 1 protein; pp65: phosphoprotein 65.

From the studied population, 61.2% (n=74) patients were positive for IE-1 specific ELISPOT assay, and 52.1% (n=63) for pp65 specific ELISPOT assay. Of the overall cohort, median values for IE-1 were 84 (IQR 5-395) spots/3x10^5^, seropositive patients’ median response to IE-1 was 108 (IQR 20-459.5) spots/3x10^5^ and for seronegative was 2 (IQR 1-5) spots/3x10^5^. In the case of pp65, global median values were 148 (IQR 23-439) spots/3x10^5^, for seropositive were 208 (IQR 72.5-479.5) spots/3x10^5^ and 3 (IQR 1-9) spots/3x10^5^ for seronegative patients (**Figures 1A, B**). Moreover, there was a moderate correlation between ELISPOT results against IE-1 and pp65 (r=0.447; *p*<0.0001) (**Figure 2**).

CMI against both peptides, pp65 and IE-1, was significantly higher in seropositive patients (*p*<0.0001). To be noted, 8 (7.69%) seropositive and 4 (23.53%) seronegative patients had values of 0 spots/3x10^5^ in IE-1 assay. In the case of pp65 assay, 2 (1.92%) seropositive and 4 (23.53%) seronegative patients had values of 0 spots/3x10^5^.

### 3.3 Agreement between CMV IgG and CMV ELISPOT assay results

**Figure 3:**
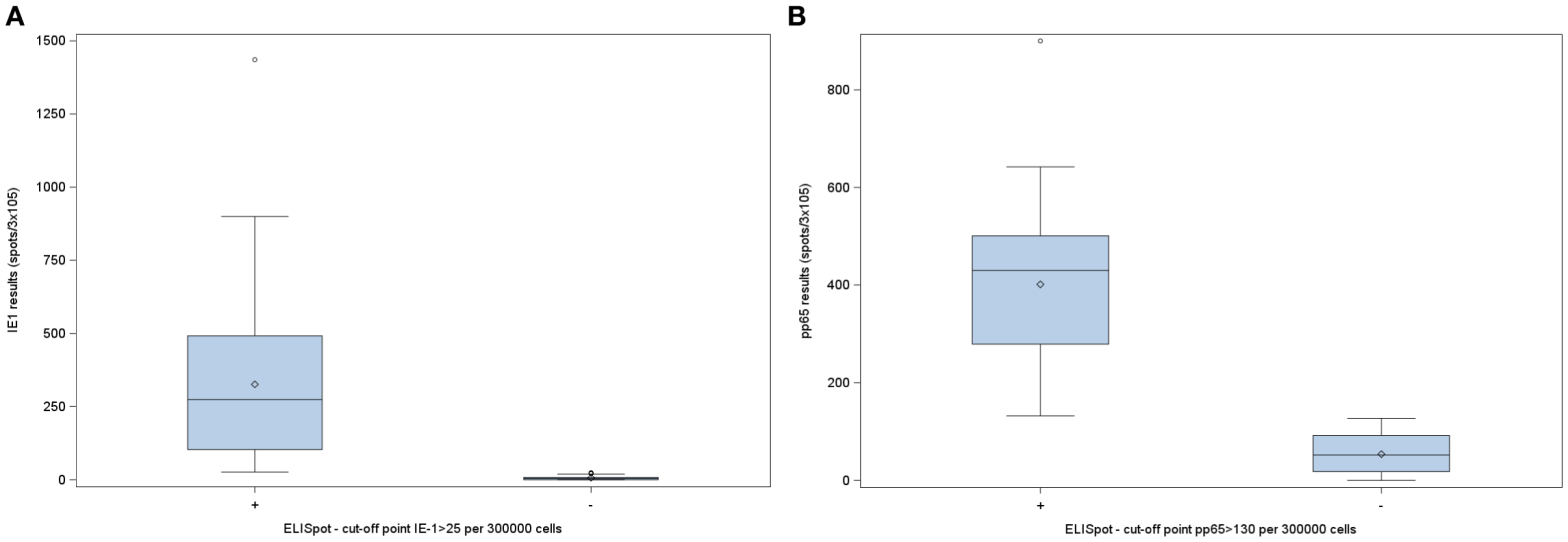
CMV ELISPOT results in CMV-seropositive patients awaiting lung transplantation. **(A)** ELISPOT responses upon stimulation with IE-1. **(B)** ELISPOT responses upon stimulation with pp65. ELISPOT, Enzyme-Linked Immunosorbent Spot assay; IE-1, immediately-early 1 protein; pp65, phosphoprotein 65; CMV, cytomegalovirus.

None of the seronegative patients was positive for any of the two CMV specific peptides by ELISPOT assay. Interestingly, 30 from 104 (28.8%) of seropositive patients were negative for IE-1 specific ELISPOT assay and 41 (39.4%) for pp65 specific ELISPOT assay (**Table 2**). **Figures 3A, B** show that from those seropositive patients there are some of them even with a null CMI response. Cohen’s Kappa agreement between both assays, IgG and CMI, was of 0.409 (95% CI 0.261-0.557, *p*<0.0001) for IE-1 and 0.302 for pp65 (95% CI 0.177-0.426, *p*<0.0001), showing a fair agreement between the two responses. In terms of percent agreement, both responses agreed on 75.2% for IE-1 and 66.1% for pp65.

**Table 2:** Categorical results of ELISPOT by serological status and percent agreement.

ELISPOT categorical results		Positive CMV IgG N=104 (86%)	Negative CMV IgG N=17 (14%)	Agreement (%)
IE-1	+	74 (71%)	0 (0%)	75.2
	–	30 (29%)	17 (100%)	
pp65	+	63 (60.6%)	0 (0%)	66.1
	–	41 (39.4%)	17 (100%)	

ELISPOT, Enzyme-Linked Immunosorbent Spot assay; IE-1, immediately-early 1 protein; pp65, phosphoprotein 65; CMV, cytomegalovirus.

### 3.4 Patient classification based on CMV-specific cellular response

When stratifying patients based on their CMV-specific cellular response determined by ELISPOT results, 40 (33.1%) were classified as having weak response, 25 (20.7%) as intermediate, and 56 (46.3%) as strong. As expected, all 17 (100%) of seronegative patients exhibited weak CMV-specific cellular response. Among seropositive patients, 56 (53.8%) were categorized as strong responders, 25 (24%) as intermediate and notably, 23 (22.1%) as weak responders (**Table 3**). Statistically significant differences were observed between the groups (*p<*0.0001). No significant differences were found in clinical and demographic characteristics between seropositive patients categorized as weak and intermediate/strong CMV-CMI (**Supplementary Table 1**).

**Table 3:** Stratification of patients based on CMV-specific cellular response using ELISPOT results and by serological status.

CMV-CMI response	Positive CMV IgG N=104 (86%)	Negative CMV IgG N=17 (14%)	p
Weak: (IE-1 ELISPOT ≤25 spots/3x10^5^ cells AND pp65 ELISPOT ≤130 spots/3x10^5^ cells)	23 (22.1%)	17 (100%)	<0.0001
Intermediate: (IE-1 ELISPOT ≤25 spots/3x10^5^ cells OR pp65 ELISPOT ≤130 spots/3x10^5^ cells)	25 (24%)	0 (0%)	
Strong: (IE-1 ELISPOT >25 spots/3x10^5^ cells AND pp65 ELISPOT >130 spots/3x10^5^ cells)	56 (53.8%)	0 (0%)	

ELISPOT, Enzyme-Linked Immunosorbent Spot assay; IE-1, immediately-early 1 protein; pp65, phosphoprotein 65; CMV, cytomegalovirus.

## 4 Discussion

Although T-cell response is known to play a key role in the control of viral infection, risk stratification for CMV infection in LT recipients is nowadays based on the assessment of the specific humoral response (21, 30, 31). In our study, discordant humoral and cellular immune responses against CMV were observed. Prior transplantation, around 30% of seropositive patients showed weak or lack of response to IE-1 and nearly 40% to pp65. In 22% of seropositive patients, there was a weak or no response to both antigens. This weak or lack of response could have implications in CMV infection risk stratification. To our knowledge, this is the first study to describe this immunological discordance in patients awaiting LT.

Previous publications showed similar results in KT. Lindemann et al. (24) compared CMV-specific humoral and CMI in 63 patients before undergoing a KT. None of the R- patients showed positive CMI, and 13 out of 39 (33%) R+ presented undetectable values. The authors suggested that in those patients with discordant results and a lack of cellular response antiviral prophylaxis after KT may be a suitable therapeutic option and proposed to study both humoral and cellular response to CMV before transplantation. Other publications also showed discordances between these two immune responses against CMV. Schachtner et al. (23) also reported similar findings in a cohort of 326 KT patients, showing that 20% of R+ patients had negative CMI. R+ patients with preformed CMI (80%) exhibited significantly lower initial and peak CMV loads, less CMV disease, reduced risk of CMV-recurrence, and less need for intravenous antiviral therapy compared to R+ without cellular activity (*p*<0.05) (23). Similarly, Lúcia et al. (22) reported that R+ had a wide range of T and B-cell responses. In particular, a non-depreciable percentage of R+ patients showed lack/weak response to IE-1 and pp65. Interestingly, Lúcia et al. (22) also observed that 25%-30% of R- presented positive CMI, which gave them greater immune protection against CMV infection after prophylaxis withdrawal compared to the R- group without cellular response. Schachtner et al. also observed in their study that although D+R- patients with a CMV-CMI did not have a lower incidence of CMV replication, they exhibited lower initial and peak CMV loads, required less intravenous antiviral therapy (*p<*0.05), and showed a tendency for less CMV disease (*p*=0.069) compared with D+R- without T cell response (23). This group of patients with discordant immune responses, being seronegative with positive CMI, did not appear in our study, perhaps due to the small proportion of seronegative patients found in our cohort.

The discordance between CMV-specific humoral and cellular response in patients awaiting LT has not been extensively studied. Cantisan et al. (32) reported that 31.8% (n=14) of seropositive patients awaiting transplantation were actually non-reactive, indicating negative CMV-CMI. However, this study combined KT (n=32) and LT (n=23) recipients, assessing CMV-CMI using a different technique, QuantiFERON-CMV (32). Another relevant study is that of Solidoro et al. (33), which assessed both humoral and cellular responses after LT. This retrospective, single-center observational study included 47 lung transplant recipients and reported a low discordance between serology and CMI, with rates of 14.2% at one month and 19% at four months after transplantation. Interestingly, they observed that all responders maintained their CMV response status over follow-up, nevertheless after 1 year all but one non-responder patients changed their status, being responders. This fact could explain the tendency to increase agreement between both responses over time from transplantation. Thus, differences between this study and ours may be explained due to differences in study design or by CMV subclinical replication after LT and its effect on the CMV specific cellular response (10). Besides, they used a cocktail mix including pp65 and IE-1 peptides to assess ELISPOT response and they set their cutoff value to 20 SFUs. Further insights are provided by Altaf et al., who assessed CMV-CMI using QuantiFERON-CMV in a cohort of 39 prospective lung transplant patients and evaluated its relationship with CMV reactivation. Interestingly, more than one-third of CMV-seropositive patients exhibited discordant cellular responses pre-transplantation. This lack of cellular response was significantly associated with a higher incidence of CMV reactivation post-transplantation, suggesting that dysfunctional CMV-specific immunity increases the risk of viral reactivation. Altaf et al. also noted that while PBMCs from these patients displayed a normal memory phenotype, they exhibited dysfunction in key memory differentiation markers, such as CD49d, which likely contributed to their impaired functional capacity (34). Additionally, Bunde et al. (35) investigated CMV-specific cellular responses in transplant recipients using flow cytometry to analyze CD4+ and CD8+ T-cell responses to pp65 and IE-1. They found that patients who did not develop CMV disease had significantly higher frequencies of IE-1-specific CD8+ T-cells (*p*=0.005). Furthermore, patients lacking a CD4+ response to one or both proteins, as well as those without a CD8+ response to IE-1, appeared to have a higher risk of developing CMV disease, although this association was not statistically significant. However, this study included only four LT patients, did not correlate findings with serology, and some results failed to achieve statistical significance. Consequently, further research involving a larger cohort of LT patients is warranted.

Risk stratification of CMV infection measured by CMV-specific cellular response prior to transplantation to adjust prophylactic treatment, has already been investigated in an international clinical trial in KT. Briefly, Jarque et al. (28) studied 160 D+/R+ stratified by their baseline CMV-CMI results and were randomized to receive preemptive or 3-month antiviral prophylactic treatment (28). The authors found that patients classified as high risk of infection (IE-1 < 20 spots/3x10^5^ PBMCs), developed significantly higher CMV infection rates than patients at low risk with both preemptive (73.3% vs 44.4%; OR, 3.44 [95% CI, 1.30–9.08]) and prophylaxis (33.3% vs 4.1%; OR, 11.75 [95% CI, 2.31–59.71]) approaches. The authors concluded that monitoring CMV-specific cellular response can help choose the most appropriate prophylaxis strategy in KT (28). It would be interesting to have a similar clinical trial in the LT field. For the time being, what is available to us is a retrospective study published by our group (26), were we observed a higher rate of CMV infection with high levels of DNAemia in patients with weak/lack of CMI response to IE-1 (cutoff point: 55 spots/3x10^5^ PBMCs), after prophylactic valganciclovir withdrawal.

QuantiFERON-CMV assay is also a widely used assay in SOT to study CMV-CMI, and has also been proposed as a potential tool to predict CMV infection after prophylaxis withdrawal (36–38) and to allow personalized CMV prophylaxis (28, 39). Manuel et al. (37) observed in a multicenter study carried out with R- and using, that patients with reactive cellular response had a lower incidence of CMV disease than patients with negative or indeterminate results (6.4% vs. 22.2% vs. 58.3%, respectively; *p*<0.001). However, in R+ and LT its usefulness is more doubtful. In the field of LT, Weseslindtner et al. (40) in a prospective study with 67 lung transplant recipients, with 39 R+, observed no statistically significant difference in CMV infection rates between patients with CMV-specific cellular response measured by QuantiFERON-CMV and those without. Westall et al. (41) used QuantiFERON-CMV to guide antiviral prophylaxis duration in LT. They reported that the incidence of CMV infection measured in bronchoalveolar lavage was significantly lower in patients who continued CMV prophylaxis until CMI was positive, compared with patients who followed standard prophylaxis. Nevertheless, there were no significant differences between groups in CMV viremia. Our group (42), in a prospective multicenter study with 86 lung transplant recipients, reported no difference in the rate of CMV infection when valganciclovir prophylaxis was withdrawn between patients with CMI measured by QuantiFERON-CMV and those without, with the rate of CMV infection around 20% in both groups. On the other hand, although combining LT and KT recipients, Cantisan et al. (32) observed a higher incidence of CMV replication in pre-transplant R+ with negative CMV-CMI. Briefly, 50% (n=7) of these patients developed CMV replication after transplantation, compared to the 13.3% (n=4) of R+ with positive CMV-CMI prior to transplantation (*p*=0.021). An ongoing clinical trial will help us clarify the role of QuantiFERON-CMV in LT (43).

Although both QuantiFERON-CMV and ELISPOT assays measure IFN-γ release, there are several differences between them. In general, QuantiFERON-CMV is easier and faster to perform than CMV-ELISPOT since there is no need to extract PBMCs. QuantiFERON-CMV measures IFN-γ production in a defined volume of blood after *ex vivo* stimulation with class I-restricted CMV peptides, while ELISPOT assay is performed on a determined number of PBMCs and allows the quantification of the number of cells secreting IFN-γ. Although there are multiple CMV-specific known proteins, pp65 and IE-1 have been identified as the predominant ones. While QuantiFERON-CMV uses 22 peptides of pp28, pp50, pp65, IE-1, IE-2 and gB, ELISPOT uses a pool of overlapping peptides that encompass all of the IE-1 and pp65 epitopes. In addition, ELISPOT allows CD4 and CD8 detection whereas QuantiFERON-CMV detects only CD8 response and is HLA type dependent (44). In terms of comparing results in clinical practice, Ruan et al. (45) recently published a systematic review and meta-analysis comparing both cellular assays and concluded that ELISPOT (using IE-1 and pp65) is a useful tool for predicting CMV infection in KT recipients, while QuantiFERON-CMV requires further investigation.

Overall, there are variable discordances between CMV humoral and cellular responses and it seems that all mechanisms involved in CMV immune response have not been elucidated yet. T-cell compartment is known to play an important role in viral replication and control (46–49) suggesting that cellular immunity can reduce or stop the extra-alveolar spread-out of CMV, but at the same time CMV produces proteins that interfere with the recognition of the virus by the immune system and its response (22, 23, 33). These mechanisms allow CMV to remain latent in healthy individuals for years and perhaps, could explain the discordance observed. Genetic variability of both host and virus could modulate CMV virulence in immunosuppressed graft recipients (50). This can confer a significant interindividual variability in immune response with a wide range of IgG titers and cellular responses against IE-1 and pp65. It is also important to note that IgG is a mere surrogate of the whole humoral adaptive immune response and that, up to now, we are excluding the memory B-cell compartment (22, 51, 52). Perhaps, studying this compartment would help us having more accurate information about these discordances. Besides, from the cellular compartment with the ELISPOT assay performed we are only assessing those PBMCs producing IFN-γ, but we are not assessing a wide range of immune cells that might be involved in controlling CMV infection.

Further studies including an exhaustive follow-up after LT are needed to accurately assess the real risk of infection and disease in R+ patients with weak or lack of CMV-CMI. Collecting samples at critical time points, such as at the withdrawal of prophylaxis—whether at 6 or 12 months per guidelines, or earlier due to adverse effects—could yield more precise and actionable data. Additionally, sampling during CMV infection episodes could provide valuable insights into how the infection modulates the CMV-specific cellular immune response.

To deepen the understanding of CMV-specific humoral responses, analyzing B-cell repertoires using flow cytometry would complement standard assessments of anti-CMV IgG serostatus. Similarly, examining T-cell populations via flow cytometry could shed light on cases of seropositive patients with weak or intermediate CMV-CMI responses, both pre- and post-LT. For instance, T-cells in these patients may exhibit signs of exhaustion, characterized by increased expression of inhibitory receptors like programmed cell death protein 1 (PD-1). Exploring both T and B-cell populations could help elucidate why some seropositive patients develop CMV infections despite varying CMV-CMI levels, while certain seronegative patients remain uninfected.

Zieliński et al. (21) published a comprehensive study in KT patients that analyzed T and B cells, natural killer (NK) lymphocytes, CD28 expression, relative telomere length, CMV-specific lymphocytes and serum cytokines. Among other interesting findings, they demonstrated that CMV promotes immune exhaustion in KT patients, with D+/R+ patients being at higher risk of CMV-associated immune senescence (21). Replicating such studies in LT patients, would represent pioneering work in understanding these immune dynamics in LT recipients. Given the unique challenges in LT, switching to a purely preemptive approach may be difficult to implement. However, conducting a clinical trial to evaluate whether CMV prophylaxis duration could be guided by ELISPOT-CMV results would be highly valuable. In conclusion, an in-depth analysis of both CMV-specific humoral and cellular responses could significantly enhance the evaluation of CMV infection risk based on individual immune profiles and potentially optimizing antiviral use while reducing unnecessary exposure to prophylactic agents.

One of the limitations of our study is the possible technical variability between the different participating centers, which we attempted to minimize by using an experienced central laboratory to perform ELISPOT assays. Thus, to reduce the variability of the results, for the specific cellular response assessment, blood samples were drawn and sent to be processed within 24 hours in the same experienced laboratory. CMV serology was determined at the clinical laboratories of each hospital, using the cutoff value of positivity that are used in the normal clinical practice.

## 5 Conclusions

According to our results, over 20% of patients awaiting LT with positive CMV serology, displayed a weak CMV-CMI response by ELISPOT-T assay. Identifying patients on the LT waiting list with seropositive CMV status but weak CMV-CMI responses, could lead to changes in the risk stratification for CMV infection after LT. Relying solely on CMV serology for risk stratification after LT may underestimate the risk for some seropositive patients. Further studies including an exhaustive follow-up after LT are needed to assess the real risk of infection and disease in R+ with weak or lack of CMV-CMI.

## Data Availability

The raw data supporting the conclusions of this article will be made available by the authors, without undue reservation.

## Supplementary material

Supplementary Table 1 Clinical and demographic characteristics of seropositive patients categorized by CMV-specific cellular response as weak or intermediate/strong.
