# Supplementary material for: Discordance between humoral and cellular immune responses to cytomegalovirus infection in CMV seropositive patients awaiting lung transplantation
Source: Front Immunol. 2025 Jan 22;15:1445553. doi: 10.3389/fimmu.2024.1445553 (PMC11794206; doi:10.3389/fimmu.2024.1445553)
Supplement: Supplementary Table 1 — Clinical and demographic characteristics of seropositive patients categorized by CMV-specific cellular response as weak or intermediate/strong. [file Table1.docx]

Supplementary Material

**Supplementary Table 1**. Clinical and demographic characteristics of seropositive patients categorized by CMV-specific cellular response as weak or intermediate/strong.

|  | **Overall (n=104)** | **Weak levels of CMV-CMI**  **(n=23)** | **Intermediate/Strong levels of CMV-CMI**  **(n=81)** | **p**^1^ |
| --- | --- | --- | --- | --- |
| Age, mean years (SD) | 57 (9.4) | 54.2 (12.3) | 57.7 (8.3) | 0.2037 |
| **Gender, n (%)** | | | | |
| Male | 64 (61.5%) | 14 (60.9%) | 50 (61.7%) | 0.9404 |
| Female | 40 (38.5%) | 9 (39.1%) | 31 (38.3%) |  |
| **Blood group, n (%)** | | | | |
| A | 27 (26%) | 9 (39.1%) | 18 (22.2%) | 0.1324^(c)^ |
| B | 13 (12.5%) | 3 (13%) | 10 (12.3%) |  |
| A/B | 3 (2.9%) | 1 (4.3%) | 2 (2.5%) |  |
| O | 61 (58.7%) | 10 (43.4%) | 51 (62.9%) |  |
| **Underlying lung disease, n (%)** | | | | |
| Interstitial lung disease (ILD) | 41 (39.4%) | 8 (34.8%) | 33 (40.7%) | 0.9131^(c)^ |
| Chronic obstructive pulmonary disease (COPD)-emphysema | 39 (37.5%) | 8 (34.8%) | 31 (38.3%) |  |
| Cystic fibrosis | 6 (5.8%) | 2 (8.7%) | 4 (4.9%) |  |
| Bronchiectasis | 3 (2.9%) | 1 (4.3%) | 2 (2.5%) |  |
| Pulmonary hypertension | 1 (1%) | 0 (0%) | 1 (1.2%) |  |
| Other | 14 (13.5%) | 4 (17.4%) | 10 (12.3%) |  |
| **Underlying conditions** | | | | |
| Hypertension | 25 (24%) | 7 (30.4%) | 18 (22.2%) | 0.4160^(c)^ |
| Dyslipidemia | 23 (22.1%) | 4 (17.4%) | 19 (23.5%) | 0.5362^(c)^ |
| Diabetes | 14 (13.5%) | 4 (17.4%) | 10 (12.3%) | 0.5049^(f)^ |
| Hepatitis B | 3 (2.9%) | 0 (0%) | 3 (3.7%) | 1.0000^(f)^ |
| Autoimmune disease | 2 (1.9%) | 0 (0%) | 2 (2.5%) | 1.0000^(f)^ |
| Hepatitis C | 1 (1%) | 0 (0%) | 1 (1.2%) | 1.0000^(f)^ |
| None | 22 (21.2%) | 5 (21.7%) | 17 (21%) | 1.0000^(f)^ |
| Other | 52 (50%) | 11 (47.8%) | 41 (50.6%) | 0.8132^(c)^ |
| Unknown | 3 (2.9%) | 2 (8.7%) | 1 (1.2%) | 0.1223^(f)^ |
| **Risk factors** | | | | |
| Current smoker, n (%) | | | | |
| Yes | 0 (0%) | 0 (0%) | 0 (0%) | 0.8429 |
| No | 38 (36.5%) | 8 (34.8%) | 30 (37%) |  |
| Former smoker | 66 (63.5%) | 15 (65.2%) | 51 (63%) |  |
| Total years smoking (only former smokers), mean (SD) | 12.8 (11.1) | 15.4 (11.2) | 12.2 (11.2) | 0.1853 |

^1^ Comparisons between groups: Mann–Whitney U-test for continuous variables; Chi-squared test for categorical variables. ^1^Comparisons between groups: Chi-square test (c) or Fisher’s exact test (f). SD: standard deviation.
